# Future Avenues for Educational Neuroscience From the Perspective of EARLI SIG 22 Conference Attendees

**DOI:** 10.1111/mbe.12211

**Published:** 2019-07-23

**Authors:** Annie Brookman‐Byrne, Lia Commissar

**Affiliations:** ^1^ Centre for Brain and Cognitive Development, Department of Psychological Sciences, Birkbeck University of London; ^2^ Centre for Educational Neuroscience University of London; ^3^ Wellcome Trust, Gibbs Building

## Abstract

The 2018 EARLI SIG 22 Neuroscience and Education conference aimed to facilitate the discussion and sharing of research and translation in educational neuroscience. In this article, we first describe and evaluate the approach taken in organizing the conference, which followed recommendations from the educational neuroscience community. We then summarize responses to a survey that captured delegates' visions of research and translation, their intentions following the conference, and the support they need moving forward. From 88 completed surveys, we first note a common desire for more discussions and collaborations across disciplines, and between teachers and researchers. We highlight particularly novel ideas that are not frequently addressed in the community so far, including discussion of ethical issues, inclusion of learners in research development, open resources for teacher training in neuroscience, and mentoring networks for community members. In sharing these ideas, we highlight future directions for the field as it continues to develop.

The European Association for Research on Learning and Instruction (EARLI) Special Interest Group (SIG) 22 Neuroscience and Education 2018 conference was hosted by the Wellcome Trust (wellcome.ac.uk) in London, UK. The Wellcome Trust's “Education and Neuroscience Initiative” aims to develop and evaluate neuroscientific evidence‐informed teaching and learning practices, and to support teachers to understand and access this science. SIG 22 aims to bring together researchers who investigate learning and development from a diverse range of fields. Together, SIG 22 and the Wellcome Trust share a desire to bring scientific research to teaching and learning. Prominent figures in the field view educational neuroscience as a new, interdisciplinary enterprise that is characterized by collaboration between researchers of different backgrounds and teachers (Howard‐Jones et al., 2016).

Here, we describe the approach taken in the organization of the conference, and consider the extent to which we were able to follow and put into practice the recommendations of delegates at a previous conference on the same theme. We also provide an overview of responses to a survey which was taken by delegates at the conference, relating to how the field might continue to move forward. The intention is to highlight good practice and learning for future conference organizers (rather than to formally analyze the survey responses) and discuss key areas for development that community members can work on together. We hope that this article will be a useful summary of the current perspectives of the educational neuroscience community. Note that in this article we refer to the field of educational neuroscience, but we currently consider this to be interchangeable with mind, brain, and education (MBE).

## ORGANIZING THE CONFERENCE

In the organization of the SIG 22 conference, we were keen to take on board the recommendations arising from a previous educational neuroscience conference. In 2016, the Wellcome Trust organized the preconference to the International Mind, Brain, and Education Society (IMBES) conference. Conversations from the day were captured and reported, resulting in particular recommendations for conference organizers (Commissar & Brookman‐Byrne, 2018). As Wellcome was involved in both conferences there was momentum to ensure the learning and feedback from the IMBES conference was put into practice at the SIG 22 conference which had similar aims. Here, we take each recommendation in turn, describe how we aimed to meet the recommendation in the organization of the SIG 22 conference, and evaluate whether our steps to meet the recommendations were successful.

1. *Carefully consider what participants should gain from attending the conference and use this aim to guide the approach and agenda for the conference*.

Taking inspiration from the education sector, for a lesson or learning experience to be effective, it is considered important to start with the outcomes you wish to achieve and knowledge of your audience's interests and level of knowledge. From experience of attending and supporting in the organization of a variety of conferences, this is not a common starting point for conference organizers. The starting point of the SIG 22 conference planning revolved around carefully defining the objectives and outcomes we intended delegates to achieve.

As with other academic conferences, we still wanted delegates to have the opportunity to hear about current research in the sector, but with the conference representing a multidisciplinary research community, we wanted to balance the research with other sessions where delegates could also hear about translation in educational neuroscience. With this in mind, we selected a range of speakers who would be able to cover both research and translation; for instance one symposium was entitled “Making science accessible to the public, teachers, and policy makers.” This differed from a more traditional approach where sharing of research is the key priority (Buddie, 2016). Since educational neuroscience aims to impact on education, we thought it was important to highlight work that has had success in this regard. In addition we gave all delegates the opportunity to not only *hear* about translation efforts, but also be involved themselves, with a number of people taking up the offer to be interviewed by the *Learning Scientists* about their work for a podcast on learning aimed at teachers (learningscientists.org/learning‐scientists‐podcast).

We also incorporated opportunities for everybody to share their research and translation experiences and ideas, not just the invited speakers (more on this below). This organizational approach led to a broad range of sessions which showcased excellence in both research and translation, and provided a platform for practitioners as well as researchers to share the progress being made in this sector, which we hope inspired others. We had positive feedback from a number of delegates with regards to the sessions presented, with one commenting that they “appreciated the variation of presenters and types of presentations.” The full conference program can be viewed online: osf.io/34vhd.

2. *Think carefully about what and how you will capture the discussions from the day and what would be the most useful outlet following the conference*.

To ensure longevity of the conference and access to those who were unable to attend in person, each talk was filmed (tinyurl.com/sig22‐videos) and all presentation slides were shared (tinyurl.com/sig22‐slides). The videos have been viewed 387 times at the time of writing, indicating that the sharing of material after the conference was worthwhile. We decided it would be valuable to collect the community's views of the future of educational neuroscience, to capitalize on the range of backgrounds and expertise present, so we carried out the survey described below (which also aimed to report interesting possible future avenues for the field). In addition, the discussions from the final day were captured through notes and photos taken by the delegates, which were collated into a report that was circulated to all delegates. This meant they were able to connect on areas of shared interest after the event, something we will discuss more below.

3. *Take an inclusive approach that acknowledges the differing backgrounds of a multidisciplinary audience and ensures the programming and content is accessible to all*.

Ahead of the event speakers were asked to plan their talks avoiding the use of jargon, defining any acronyms, and ensuring their talks were accessible for a range of backgrounds. At the opening of the conference it was again emphasized to all delegates that “everyone in attendance was an expert in their field, but that many attending had different expertise” and that asking questions for clarification was encouraged to ensure everyone had a shared understanding of the topic being discussed. We received feedback from a number of attendees that this made them feel more comfortable in asking questions of others from different fields.

We felt it important during the planning to acknowledge that delegates were coming with different expertise, from multiple countries, and with different levels of knowledge about the field. In an attempt to support conversations during the event, those attending were provided with reading material in advance, to enable them to come to the conference with some shared basic background knowledge of the field. For future conferences, we think more could be done to ensure everyone starts the conference with some shared knowledge, such as a short talk that summarizes the field, since it is likely that not all delegates read the background material that was distributed. Another option could be a preconference for those new to the field which would provide a more historical and fundamental introduction to the field. In addition, conference organizers could organize for a person to sit close to the front and hold up a card to indicate to a speaker (in real time) when jargon or country‐specific language had been used, so that they could further clarify it during their talk.

4. *Adopt more practically led, discussion‐based approaches that encourage delegates to share their expertise and to think about future directions of the field*.

The final morning of the conference consisted of an OpenSpace format event that enabled delegates to work on topics, issues, or projects important to them, as the agenda was entirely constructed by those in attendance on the day. This gave all delegates the opportunity to use their shared expertise to work with others to discuss ideas and projects; to make progress in the field. The OpenSpace format was organized around the question “what can we all do to work together better and improve learning?”; a purposefully open question, to allow all delegates (researchers and practitioners) to feel able to contribute. By dedicating the final half‐day of the conference to this format it ensured everyone left the conference having had the opportunity to discuss the topic of most interest or importance to them, and to meet others also interested in developing that area.

The format was professionally facilitated by external experts who created a climate of inclusivity and equality, highlighting the need for open discussions without hierarchy. Delegates were invited to pose their own topics for discussion, and those who did so inserted their discussion topic into a wall chart to indicate where they would be holding their discussion, and at what time, so that others could choose which discussions to attend. No discussions were decided in advance, and all topics were chosen by delegates. There were three separate sessions, within which over 45 different conversations relating to theory, research, and translation in the field took place. Delegates were encouraged to move freely between discussion groups and join whichever conversation they wanted to, ensuring that no one was caught in discussions which they did not feel they could meaningfully contribute to or learn from. This format enabled conversations across disciplines and between researchers and teachers. Those who led a discussion wrote a summary, and these were collated into a report that was later distributed to all delegates.

At the end of the event, a microphone was passed round to all delegates, so that everyone had the opportunity to share a key thought or message from the day. Many delegates expressed how they had felt unsure about the format of the OpenSpace before they attended, but that it surpassed their expectations and was a valuable opportunity to connect to colleagues and get new ideas for projects started. Dedicating a large portion of the conference to discussion was a great opportunity for everyone to have the chance to think about their next steps.

5. *Where a conference aims to include educators, ensure the program is relevant and accessible, and make efforts to invite teachers, offering financial help if possible*.

The conference aimed to include educators, in order to learn from their expertise and to share information that might be of use to them. Teachers were invited through emails and social media, and a discounted price was offered for teachers. We also hope that any interested teachers who were unable to make the conference will access the videos and slides online. A challenge was ensuring that the material presented was accessible to all. While speakers made efforts to use clear language, it is likely that there were nonetheless aspects of the conference that were difficult for all delegates to access. The language barrier remains an ongoing issue in the field (Ansari & Coch, 2006; Commissar & Brookman‐Byrne, 2018), and future conferences could provide a list of common words, phrases, and acronyms to enhance accessibility. In addition, one aspect which we did not ask speakers to specifically consider when giving talks, was the country‐specific terminology used in education, which in any future events we would like to encourage speakers to explain more simply (for example, referring to age rather than grade).

Overall, we found these recommendations useful in guiding the organization of the conference. Enabling plenty of discussion among delegates was a priority, and we received positive feedback that this was appreciated by those present, many of whom had never attended a similar OpenSpace event. Despite some initial reservations at this uncommon approach, delegates enjoyed the opportunity to talk about their own passions and move projects forward with help from other community members. A second priority in organizing the conference was to provide a snapshot of delegates' views around what the field needs in order to progress, which we discuss further below.

## DELEGATES' VISION OF RESEARCH AND TRANSLATION

During the conference, a survey designed to capture community members' vision of future avenues in research and translation in educational neuroscience was distributed to delegates. The procedure received ethical approval from the local ethics committee, and the full survey can be found online: osf.io/34vhd/. Paper surveys were distributed in the conference lecture theater at the end of the second conference day, and delegates were asked to complete the survey there and then. We received responses from 88 of 124 delegates at the conference, who were asked to indicate their role from six categories. Thirty‐three delegates chose more than one category, with 13 describing themselves as teachers, 39 as psychologists, 20 as educational scientists, 44 as neuroscientists, 0 as geneticists, and 12 as “other.” Those who identified as researchers indicated their level: 33 were postgraduates, 4 were research assistants, 12 were postdocs, 12 were lecturers or senior lecturers, 15 were readers or professors, and 5 were teacher–researchers. When asked how much they know about the field of educational neuroscience, 17 reported that they knew not very much, 47 reported that they knew a fair amount, and 23 reported that they knew a lot. There was therefore a range of backgrounds, levels, and prior knowledge represented at the conference and in the survey, emphasizing the importance of ensuring no assumed shared background knowledge throughout the conference.

The key survey items of interest were: (1) My vision for research in the field of educational neuroscience is… (2) My vision for translation in the field of educational neuroscience is… (3) Following the conference I intend to… and (4) This is the support I need… Many of the responses referred to the ongoing need for communication and collaboration between teachers and researchers, and across scientific disciplines, with 30 responses referring to communication and/or collaboration. Since the requirement of increased communication and collaboration between sectors has been well documented (e.g., Ansari & Coch, 2006; Brookman‐Byrne & Thomas, 2018; Varma, McCandliss, & Schwartz, 2008) we will not elaborate further on this point. However, this was a theme picked up and further discussed by some delegates on the final day. Therefore, there is clearly still a need within the sector for funding or organizational structures to support this. Instead we will pull out less well‐versed suggestions and thoughts that we think are novel, with the potential for moving the field forward, and where there are particular action points for the community to work on together. The four questions were used to stimulate thoughts and responses about the future of the field in terms of both personal views and practical suggestions. Rather than addressing each question in turn, our approach was to focus on responses that we thought the community would benefit from, regardless of which survey item they appeared under.

## ETHICS IN EDUCATIONAL NEUROSCIENCE

A few delegates referred to ethics, morals, and values within educational neuroscience. This topic is not often discussed in the field, with a brief mention in the Royal Society (2011) Brain Waves report; specifically that ethical issues relating to access and fairness need to be taken account of in research, in particular when considering cognitive enhancers such as nootropic drugs. Since these are increasingly taken by those with no specific need (rather than those with diagnosed developmental disorders, who the drugs were intended for), ethical issues arise (Sahakian & Morein‐Zamir, 2007). Beyond important questions around their safety and efficacy, unequal access to these drugs could lead to unfair disadvantages for those who do not take them, for example if poorer students are unable to afford them, and consequently perform less well in an exam than those who can afford them (see Bostrom & Sandberg, 2009; Sahakian & Morein‐Zamir, 2007 for more in‐depth discussions).

While ethical discussions in the field have mostly been confined to conversations about cognitive enhancers and brain stimulation (Cohen Kadosh, Levy, O'Shea, Shea, & Savulescu, 2012), there are other important areas for debate. During the conference, there was a keynote from Professor Robert Plomin on genetics in education. This particular topic leads to important ethical questions, and indeed one delegate was keen to receive information about the ethics concerning genetics, which may have been prompted by Plomin's talk. Given the likelihood of a move toward the use of genetics for personalized education (Hart, 2016), it is essential that there are conversations about how this individual genetic information is used. It is anticipated that this information would lead to tailoring of education to specific individual needs (Hart, 2016), but both public discourse and policy making are necessary to ensure that the involvement of genetics in education is positive, and that interventions are not applied ahead of the evidence.

One delegate called for discussion and critical perspectives around morals and values in the field more widely. Discussion around the ethical issues arising from educational neuroscience does appear to be gaining momentum, as a recent review of the field spoke of the need to address new ethical issues arising (Thomas, Ansari, & Knowland, 2018). This review pointed to potential concerns surrounding predictive measures of educational outcomes, particularly since children cannot give their consent, and there is the possibility that such predictive measures would influence the child's education or future. It is clear that there is both increasing appetite and need to discuss ethical issues in educational neuroscience, particularly as more sophisticated technologies are applied to education. Forums for such discussion are therefore an action point for the field moving forward, and these conversations would need to include those outside of the community who will also have views on how children's information should be used, as well as learners, teachers, and those representing them.

## WORKING WITH STUDENTS

While there is often discussion around the need to collaborate with teachers, a less common discussion concerns the need to collaborate with learners themselves. Three delegates suggested that there should be efforts to engage with students. One suggested developing students' understanding of the science of learning, and efforts to do this have begun, such as the *Learning Scientists* blog (learningscientists.org), the book *Learning How to Learn* (Oakley, Sejnowski, & McConville, 2018), and the journal *Frontiers for Young Minds* (kids.frontiersin.org), which is aimed at children and includes topics within educational neuroscience (e.g., Turoman, Merkley, Scerif, & Matusz, 2017). Nonetheless, there is clearly still more to be done to enable all students to access key information. As more educators become involved in the field and access the increasing online resources, science relating to learning will likely trickle down to learners through that avenue.

Another delegate suggested finding out what students require in order to effectively use results from educational neuroscience research. This is an important step since raising awareness with students does not necessarily lead to change in behavior (Cain, Gradisar, & Moseley, 2011). Perhaps what is needed is a program of research into the best way to present this information that would lead to students altering their learning strategies effectively. However, it is likely that there will be individual differences in how students respond to the information, so a range of methods of getting information to students may be a good step for now. In addition to the aforementioned blog, book, and journal, these methods could include school visits from researchers, podcasts, interactive websites, games, and videos.

Finally, another delegate suggested conducting research that involves cocreation with students, which perhaps would involve speaking with students at the beginning of a research project, in order to incorporate their views, or gathering ideas from students about what they think researchers should be investigating; guiding the research based on the needs of students. This would differ from a more traditional narrative in educational neuroscience which is for the needs of teachers to be a primary guiding force (Brookman‐Byrne & Thomas, 2018). Engaging with students in this manner has the potential to change the focus of educational neuroscience research: for example, away from simply maximizing learning and improving test performance, toward reducing stress when learning, or helping students to identify their strengths and good career options. Given the proposed greater involvement of ethics conversations in educational neuroscience, it could be argued that including students in this manner is a more ethical approach, in doing more to take into account the needs of those affected by the research. It is clear that educational neuroscience could have a role in researching and potentially combating school‐related stress, since both neuroscience and psychology have contributed to the science of stress (e.g., see a review paper by Wolf, 2009, on the link between stress hormones and long‐term memory). It is therefore also possible that focusing on student‐led questions outside of learning might have a knock‐on positive impact on school performance; a side effect of reduced student stress may be increased learning. A student‐oriented approach could lead to exciting new avenues.

## TEACHER TRAINING

In addition to general communication between researchers and teachers, a number of delegates were especially keen for more educational neuroscience information to reach teachers through initial teacher training (ITT) and continuing professional development (CPD). These suggestions may have been inspired by a keynote from Professor Paul Howard‐Jones, who described his efforts to incorporate the science of learning into secondary school ITT (edneuro.net). In this University of Bristol ITT program, the team started from the perspective of what is considered good education pedagogy, and then helped teachers to understand from a neuroscientific perspective why those practices may be effective. The team grouped relevant research into the three broad categories: engagement, building, and consolidation (Howard‐Jones et al., 2018). The ITT explores the subcortical structures involved in *engagement* of the learner, working memory systems involved in the *building* of new knowledge, and the shifting of information from working memory to regions that enable long‐term *consolidation* (Howard‐Jones et al., 2018). By using research to underpin the teachers' understanding of learning, the aim was to empower them to tweak their practices to be able to reflect on why sometimes they were not working well, and how to make them more effective. The University of Bristol's teacher training provision was inspected by Ofsted (the Office for Standards in Education, Children's Services and Skills in England) during the integration of this research into their ITT provision, and it was recognized as part of their outstanding training for teachers: “The University of Bristol is a research‐led institution. Research, such as in neuroscience and cognitive psychology, is an integral part of the training programme. Subject‐based teaching and pedagogy is informed by internationally recognised research and used exceptionally well by trainees to develop their teaching.” This demonstrates a wider recognition from the education system of the value of integrating educational neuroscience research into teacher training.

Efforts to bring the science of learning to teachers are also currently being trialed in a primary setting (McMahon, Etchells, & Yeh, 2018), where an interdisciplinary team of researchers in psychology, education, and neuroscience are developing materials to help teachers in both supporting children's learning, and assessing scientific evidence (McMahon & Etchells, 2018). Headway is therefore being made with regards to bringing the science of learning to teacher training in the United Kingdom, although we are still a long way from having this information across all ITT programs.

Two delegates requested advice from the community about what works in teacher training sessions, and how to go about creating a workshop for teachers, for example in terms of format. In particular, one delegate asked what makes a good CPD session for teachers, highlighting that there may be keen researchers who want to pass knowledge on but do not possess an adequate understanding of what is required. In England, there is a general standard for quality CPD (Department for Education, 2016), but less is known or has been shared about what works specifically with content on the science of learning. This is an action point for the community: can resources and materials be shared so that scientists know how to give an appropriate teacher training session? A related question is: how can scientists let schools know that they are keen to help out in this manner? As ever, greater communication between schools and teachers will help this process, but as yet there is no place for each to find the other. During the OpenSpace session, one group of delegates discussed setting up a matchmaking service for schools and researchers to find each other, discussions about which are ongoing.

## BUILDING NETWORKS

Supportive networks within the field were suggested. One delegate intended to build a network of teacher educators to explore how practices are changing. This network would likely be an online forum or email list, to enable discussions to occur internationally, and without too much work to set up. Teacher educators could work together to consider how the latest scientific evidence relates to theories of education; going beyond applying the science of learning to practice, and integrating it into educational theory. The building of such a network could tie in with the creation of accessible resources for teacher training, ensuring that everyone has access to the latest science and related useful materials.

There was also the suggestion of supportive advisor and peer networks. In terms of peer networks, the IMBES Trainee Board has a private Google group for student and postdoc members of the community. There are also Twitter conversations about the field which can be found through the hashtag #edneuro. We are unaware of any other efforts to connect those in the field, other than conferences, and there does appear to be a particular lack of schemes for mentoring networks. While there are likely many informal mentoring relationships, an action point for the field could be to set up formal networking opportunities. This could be through appointing senior mentors to regularly meet with junior researchers who do not already work together, to ensure that the junior researcher can be open about their goals and concerns. As educational neuroscience is still a relatively small field, these networks have the potential to help community members feel supported.

## LESSONS FOR FUTURE CONFERENCES

Given the visions and potential avenues presented above, there are features that could be implemented in future conferences to help members of the community in moving toward these visions. A conference session on ethics or genetics would enable the discussion of key ethical concerns arising from educational neuroscience research, and could include a parents' view, since parents are likely to have strong views about aspects of this topic. To address the involvement of students in the design and dissemination of research, a school local to the conference venue and with contacts within the organizing committee could be approached. Keen students could attend a portion of the conference, perhaps presenting their view on what they'd like to know about the science of learning, or engaging in small group discussions with researchers and teachers. This also has the potential to be a good learning opportunity for students who are interested in a career in science, which might help to sell the idea to school staff.

In order to move forward with regards to incorporating the science of learning into teacher training (both ITT and CPD), future conferences could include more sessions that present how different programs are doing this. To move these efforts forward, there could also be discussion groups for those who are keen to develop materials and content on this topic. Finally, conferences are the ideal place to encourage further mentoring and networking. Networking events could be organized, particularly for early career researchers who might benefit from greater contact with others at a similar career stage (see the 2018 IMBES trainee preconference for a great example of this kind of event). A conference could also be a good place to start advisory mentorships between senior and junior researchers, perhaps through a networking event where those who are interested can meet, and arrange to keep in regular contact. Overall, we are convinced that the key principles for conference organization are to allow for plenty of discussion and sharing of ideas, and capturing those conversations so that all members of the community can gain.

Where conferences are hosted and planned by different organization members each time, more should be done to pass on learning and views from the sector, so that conferences evolve; delivering content and opportunities required by the sector.

## CONCLUSIONS

The EARLI SIG 22 conference aimed to follow the recommendations of the educational neuroscience community to ensure effective sharing of research and translation in the field. We prioritized discussion between experts, and the capturing of experts' views through sharing conference resources, and survey distribution. The overwhelming sense from the survey was that community members envision further growth in collaboration and communication across disciplines and crucially between educators and researchers. Community members also called for a focus on ethics within the field; with the sometimes controversial involvement of genetics and cognitive enhancement in education, conversations surrounding ethics will be essential. Delegates saw a need for engaging directly with students, both to communicate scientific findings to learners, and to collaborate with students on research projects. The inclusion of neuroscience in teacher training was viewed positively by responders, who also requested the sharing of resources relating to teacher training. Finally, there was appetite for strong networks to be built, including mentoring systems to provide support for less senior researchers. It is interesting to note that these suggestions from community members do not relate to research per se, rather they relate to wider issues, translation, and personal support. We hope that these suggestions will be considered by the whole educational neuroscience community, so that we can work together to move the field forward in the best possible direction.
